# Recurrent, Delayed Hemorrhage Associated with Edoxaban after Deep Brain Stimulation Lead Placement

**DOI:** 10.1155/2013/691840

**Published:** 2013-01-13

**Authors:** Walavan Sivakumar, Sarah T. Garber, Lauren E. Schrock, Paul A. House

**Affiliations:** ^1^Department of Neurosurgery, Clinical Neurosciences Center, The University of Utah, Salt Lake City, Utah, UT 84132, USA; ^2^Department of Neurology, Clinical Neurosciences Center, The University of Utah, Salt Lake City, UT 84132, USA

## Abstract

Factor-Xa inhibitors like edoxaban have been shown to have comparable or superior rates of stroke and systemic embolization prevention to warfarin while exhibiting lower clinically significant bleeding rates. The authors report a case of a man who presented with delayed, recurrent intracranial hemorrhage months after successful deep brain stimulator placement for Parkinson disease while on edoxaban for atrial fibrillation. Further reports on the use of novel anticoagulants after intracranial surgery are acutely needed to help assess the true relative risk they pose.

## 1. Introduction

Pharmacologic anticoagulation for stroke and systemic embolization prevention in the setting of nonvalvular atrial fibrillation has undergone numerous iterations in recent years. Until recently, the mainstays of therapy included platelet function inhibition, with aspirin and clopidogrel, and vitamin K blockade, with warfarin [1]. Although effective at altering thrombus formation, with a relative stroke risk reduction of 60%, these methods have been beset by numerous obstacles, which have prompted investigators to search for other methods of anticoagulation [2–4]. Recent attempts have focused on more direct coagulation factor inhibition. Large, multicenter, randomized controlled trials have shown direct factor inhibitors to be at least comparable to warfarin in regards to stroke and systemic embolism prevention while having lower overall rates of significant hemorrhage [2–4]. Unfortunately, in the event of intracranial hemorrhage in a patient on one of these newer-line agents, there are currently no effective reversal protocols in place [5].

We present a case of a patient with Parkinson disease who developed a recurrent intracranial hemorrhage months after deep brain stimulator (DBS) lead placement while being treated for atrial fibrillation with edoxaban, a novel factor-Xa inhibitor in phase III clinical trials. His unusual clinical course illustrates a problem neurosurgeons are likely to face with increasing frequency.

## 2. Case Presentation

**Figure 1:**
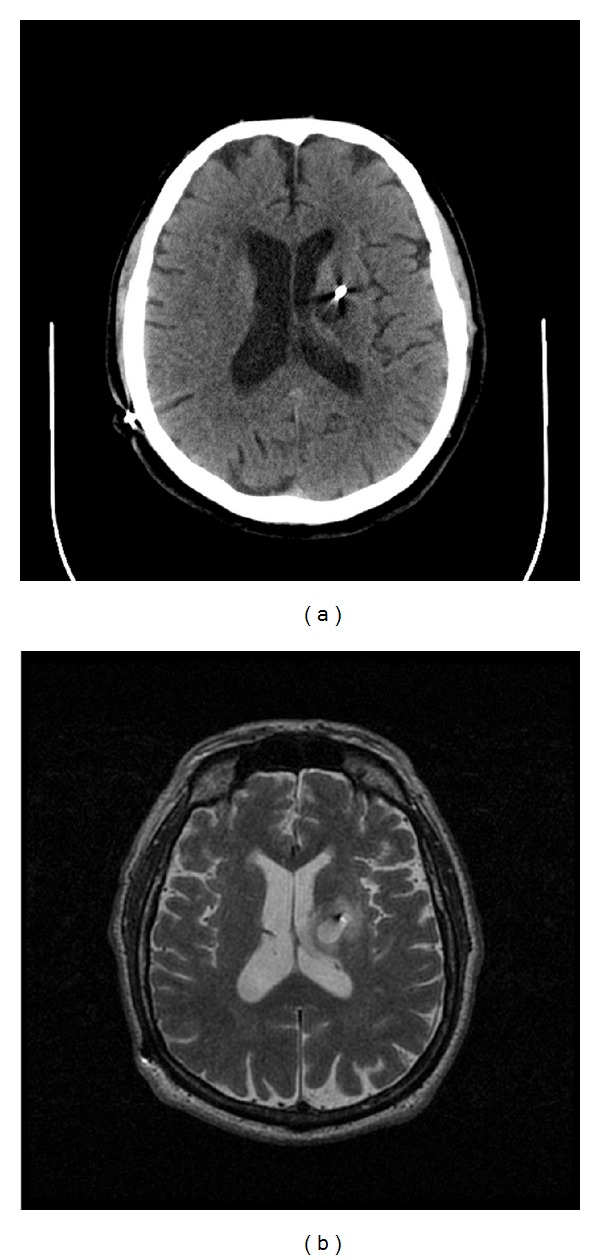
(a) Axial CT without contrast enhancement showing hypodense 1.5 cm ovoid lesion in left basal ganglia surrounding deep brain stimulator lead four months after DBS placement. (b) The lesion appears hyperintense on axial T2-weighted MRI.

**Figure 2:**
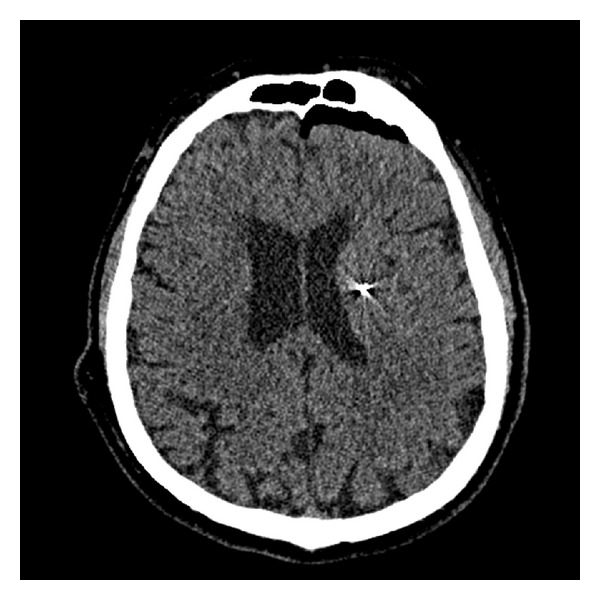
Axial CT without contrast enhancement showing no acute hemorrhage along DBS tract immediately after DBS placement.

A 72-year-old man with Parkinson disease was found to have experienced a presumed intracranial hemorrhage approximately 4 months after an uneventful placement of a unilateral deep brain stimulation (DBS) lead into the subthalamic nucleus for treatment of Parkinson disease. He presented for evaluation of short-term memory difficulties and expressive aphasia. Imaging studies (Figure 1) revealed a cystic cavity midway along the DBS lead most consistent with an intracranial hemorrhage that was resolving. Immediate postoperative imaging at the time of lead placement had not suggested any hemorrhage or infarction (Figure 2), and the patient's early postoperative course was otherwise unremarkable, with improvement of contralateral motor fluctuations and tremor and reduction of bradykinesia and rigidity. The patient did not experience an acute decline in function associated with this imaging finding but rather experienced a gradual worsening of memory and speech. It is presumed that a small hemorrhage occurred and that edema associated with the development of a hemorrhage capsule led to his delayed presentation.

**Figure 3:**
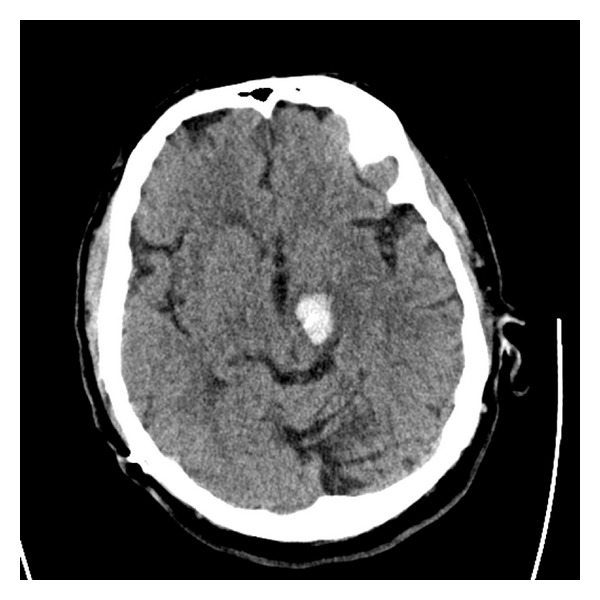
Axial CT without contrast enhancement showing 1.9 × 1.5 cm acute hemorrhage in the left cerebral peduncle five months after DBS placement.

Conservative treatments including speech therapy were initiated, and his memory difficulties began to slowly improve. While recovering from the aphasia, he presented approximately one month later with the acute onset of profound dysarthria, confusion, right facial weakness, and diplopia. A noncontrast computed tomography scan showed a 1.9 × 1.5 cm acute hemorrhage in the left cerebral peduncle just distal to the tip of the DBS electrode (Figure 3). On examination, the patient was oriented to name only and was dysarthric but able to follow commands in all four extremities. All laboratory values were within normal limits, and no platelet products or clotting factors were administered.

Only during admission for this second intracranial hemorrhage it was established that the patient had continued in a randomized clinical trial of a new direct factor-Xa inhibitor, edoxaban, for atrial fibrillation. Previously, the patient had reported that he would be finished with the clinical trial one week before DBS placement. In fact, he restarted this medication only 5 days after lead placement. Unfortunately, the study participation was not revealed despite multiple medication reviews at the time of the initial memory difficulties. 

**Figure 4:**
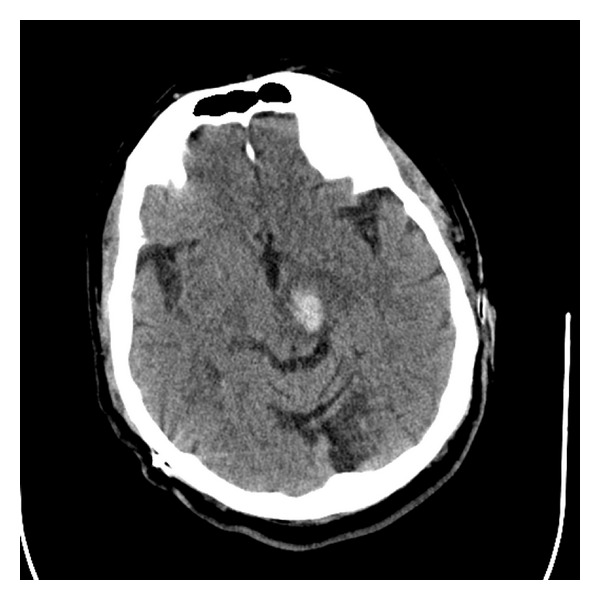
Repeat axial head CT without contrast enhancement done 24 hours after initial CT scan showing interval improvement of left cerebral peduncle hemorrhage.

The patient was admitted to the neurocritical care unit for frequent neurological examinations. A repeat non-contrast CT ~24 hours after admission showed mild decrease in the size of the hemorrhage (Figure 4). Over the next few days, the results of the patient's neurologic examinations slowly improved, although he remained intermittently confused. He was discharged to the inpatient rehabilitation unit on hospital day 7 with a plan to remain off all anticoagulation therapies. The patient was withdrawn from the edoxaban trial and was started on antiplatelet therapy after three months. He recovered well, with mild expressive aphasia and weakness; however, his contralateral bradykinesia and rigidity did again worsen as the hemorrhage resolved. 

## 3. Discussion

This paper illustrates an example of delayed, recurrent, spontaneous intracranial hemorrhage in a patient on a new irreversible, direct factor-Xa inhibitor. With the increasing use of newer anticoagulation agents in patients with atrial fibrillation, the occurrence of such events may increase. It is incumbent on neurosurgeons to document the occurrence of such events and develop management protocols.

Recent studies have shown that even subclinical atrial fibrillation can place a person at a significantly increased risk of stroke [6]. The last two decades have witnessed a robust interest in new anticoagulation agents. Agents such as fractionated/low-molecular-weight heparin and direct thrombin inhibitors provided advantages over warfarin but had limited use because of their parenteral administration [7]. Therefore, while oral vitamin K antagonists have many disadvantages, they remained the mainstay of therapy in patients requiring chronic anticoagulation. Newer direct factor-Xa (e.g., rivaroxaban, apixaban, and edoxaban) and thrombin (e.g., dabigatran) inhibitors, however, demonstrate comparative efficacy and decreased major bleeding rates compared with warfarin while also being amenable to oral administration [2–4, 8].

Edoxaban is one of three new direct factor-Xa inhibitors currently in phase 3 clinical trials for the prevention of stroke in patients with nonvalvular atrial fibrillation and the prevention of systemic embolization [2, 4, 9]. Edoxaban has been shown to be an effective inhibitor of factor-Xa because it has a 10,000-fold greater affinity for the receptor than thrombin without other relevant inhibitory effects [10]. Bolstered by comparable or lower bleeding rates than warfarin in phase II trials that appear dose dependent, the phase III ENGAGE AF-TIMI 48 trial is currently underway comparing edoxaban 30 mg once daily, edoxaban 60 mg once daily, and dose-dependent warfarin in primary stroke prevention. 

Other direct factor-Xa inhibitors have shown promising results in regards to intracranial hemorrhage. In the event of intracranial hemorrhage, however, there are no data on the morbidity and mortality surrounding the bleed or discussion of effective reversal agents such as exist for warfarin [5, 11]. 

As DBS has been used in over 80,000 patients and perioperative hemorrhage events are well documented, it seems delayed hemorrhage is likely a rare event. A recent PUBMED review of the English literature on this topic revealed only a single case of reported delayed hemorrhage with aspirin being identified as the likely causative agent [12]. To our knowledge, the current report is novel in that the hemorrhage was recurrent and in the setting of edoxaban use. 

Only general guidelines are available to help the neurosurgeon in regards to resumption of anti-platelet or anticoagulation therapy after trauma or intracranial procedures [13]. When used for stroke prevention in the setting of atrial fibrillation, anti-coagulation is routinely reinitiated in our DBS practice 6–12 weeks after elective surgery. 

The use of novel direct anti-coagulants will certainly increase rapidly in the near future as these agents are, in general, proving to be safer than warfarin therapy. Given the rarity with which delayed hemorrhage usually occurs following DBS surgery, however, it is extremely concerning that edoxaban might pose a significant risk in this particular patient population. Further reports on the use of novel anti-coagulants after intracranial surgery are acutely needed to help assess the true relative risk they pose.
